# Platelet Lysate: The Better Choice for Jaw Periosteal Cell Mineralization

**DOI:** 10.1155/2017/8303959

**Published:** 2017-12-17

**Authors:** Yvonne Wanner, Felix Umrath, Marc Waidmann, Siegmar Reinert, Dorothea Alexander

**Affiliations:** ^1^Department of Oral and Maxillofacial Surgery, University Hospital in Tübingen, Tübingen, Germany; ^2^Institute for Clinical and Experimental Transfusion Medicine, University Hospital in Tübingen, Tübingen, Germany

## Abstract

Previously, we demonstrated a high quality of minerals formed by serum-free cultured jaw periosteal cells (JPCs) by Raman spectroscopy but the mineralization extent was not satisfactory. In the present study, we analyzed the proliferation and mineralization potential of human platelet lysate- (hPL-) cultured JPCs in comparison to that of FCS-cultured JPCs. By cell impedance measurements, we detected significantly higher population doubling times of PL-cultured JPCs in comparison to FCS-cultured JPCs. However, this result was not based on lower proliferation activities but on diminished cell sizes which JPCs develop under PL cultivation. The measurements of the metabolic activities clearly showed significantly higher cell proliferation rates under PL culturing. Equivalent levels of the mesenchymal cell markers CD29, CD45, CD73, CD90, and CD105 were detected, but there were significantly increased MSCA-1 levels under PL cultivation. While JPCs only occasionally mineralize under FCS culture conditions, the mineralization potential was significantly stronger under PL cultivation. Moreover, in 4 of 5 analyzed patient cells, the addition of dexamethasone was proved no longer necessary for strong mineralization of PL-cultured JPCs. We conclude that *in vitro* cultivation of JPCs with platelet lysate is a suitable alternative to FCS culture conditions and a powerful tool for the development of high-quality TE constructs using jaw periosteal cells.

## 1. Introduction

In order to make clinical applications of tissue engineering constructs safe, we established serum-free culture conditions and observed an earlier but weaker mineralization potential of serum-free cultured JPCs [1]. By Raman spectroscopy, we identified and emphasized the differences in the biochemical composition of crystals formed extracellularly under FCS-containing and FCS-free cultivation of JPCs [2]. The diminished extent of JPC calcification as well as the significantly decreased collagen production might lead to an unsatisfactory bone formation significantly countering the success of future tissue engineering applications using this cell type.

From the beginning of the cell culture technique up to the present day, the use of fetal calf serum still represents the gold standard for *in vitro* cell cultivation. However, the development of the relatively young field of tissue engineering including the innovative 3D bioprinting and microfluidic approaches cause a long-term change of standard *in vitro* cell culture techniques/media.

In the meantime, a variety of companies provide serum-free cell culture media; however, the cultivation of some primary cells with these media is not trivial. In general, coating of the culture dishes is required for sufficient cell adhesion, the production of extracellular matrix by serum-free cultured cells is normally diminished, and lower cell densities can be achieved. As mentioned before, serum-free cultured JPCs show a reduced mineralization potential, an observation that can partially be explained by the alteration of extracellular matrix formation.

In recent years, the use of human platelet lysate has been taken into consideration as a suitable alternative to FCS, circumventing the problem of transmission of animal components and/or triggering of immune responses during cell therapies. During PL manufacture, platelets are lysed in order to achieve the release of growth factors from platelets' alpha granules [3]. After the apheresis and filtering procedures, cell debris, and leucocytes will be removed [3–5].

In order to evaluate the suitability of human platelet lysate for the in vitro culturing and osteogenic differentiation of JPCs, we analyzed in the present study the proliferation and mineralization capacity of these cells under PL and FCS culture conditions. For proliferation analysis, two completely different approaches were performed: on the one hand, population doubling times were determined by measurements of electric impedance, and on the other hand, measurements of the metabolic cell activities were carried out. Additionally, cell differentiation experiments were performed and mineralization capacities as well as mesenchymal stem cell marker expression by PL- and FCS-cultured JPCs were analyzed and quantified.

## 2. Material and Methods

### 2.1. Cell Isolation and Culture of JPCs

JPCs derived from 5 donors were included in this study in accordance with the local ethical committee (approval number 194/2008BO2) and after obtaining written informed consent. The jaw periosteal tissue was cut in small pieces with a scalpel and enzymatically digested with type XI collagenase (1500 U/ml, Sigma-Aldrich, Steinheim, Germany) for 90 min. Enzymatically isolated cells were expanded in DMEM/F12 + 10% fetal calf serum (FCS) for up to 4 passages until used in passage 5 for all differentiation and proliferation comparative assays. JPCs were cultured in different well formats depending on the used method. For 96 well plates used for MTT assays and E-plates used for xCELLigence measurements, a cell density of 2000 cells per well was chosen. For differentiation experiments, 6-well plates with a starting density of 4 × 10^4^ cells/well were used. For flow cytometric analyses of surface antigen expression, JPCs were grown in 75 cm^2^ culture flasks with a starting density of 5 × 10^5^ cells/flask. JPCs were cultured in DMEM/F12 (Invitrogen-BioSource Europe, Nivelles, Belgium) containing 10% FCS (Sigma-Aldrich, Steinheim, Germany) or 10% platelet lysate containing 1% amphotericin B and penicillin/streptomycin (Biochrom, Berlin, Germany). The used PL was provided by the Institute for Clinical and Experimental Transfusion Medicine in Tübingen, did not contain heparin, and was referred to as a research lysate based on the absent quarantine period. DMEM-cultured cells were passaged using trypsin-versene EDTA (1x, Lonza, Basel, Switzerland), and medium change was performed three times per week.

**Figure 1:**
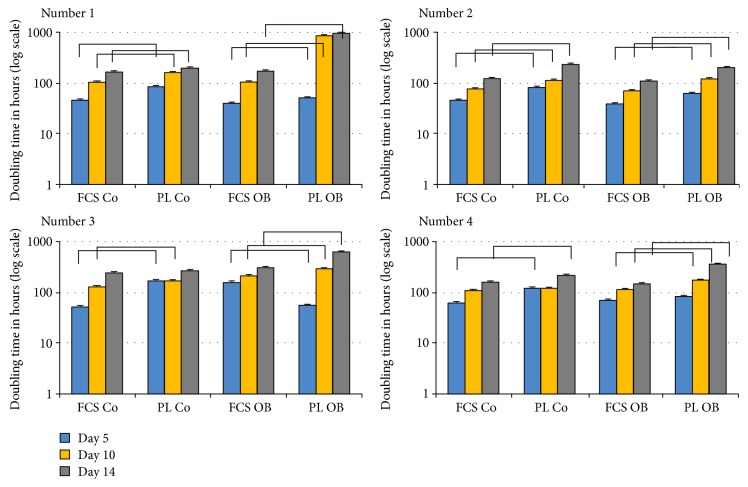
JPC population doubling times using the live-monitoring system xCELLigence. JPCs from 4 donors (numbers 1–4) were cultivated under FCS and human PL supplementation under normal (Co) and osteogenic (Ob) conditions for 5, 10, and 14 days in E-plates for electrical impedance measurements. Population doubling times (in hours, logarithmic scaling of the *x*-axis) were calculated by the device-specific RTCA software. The bars indicate statistical significances: *p* < 0.05.

**Figure 2:**
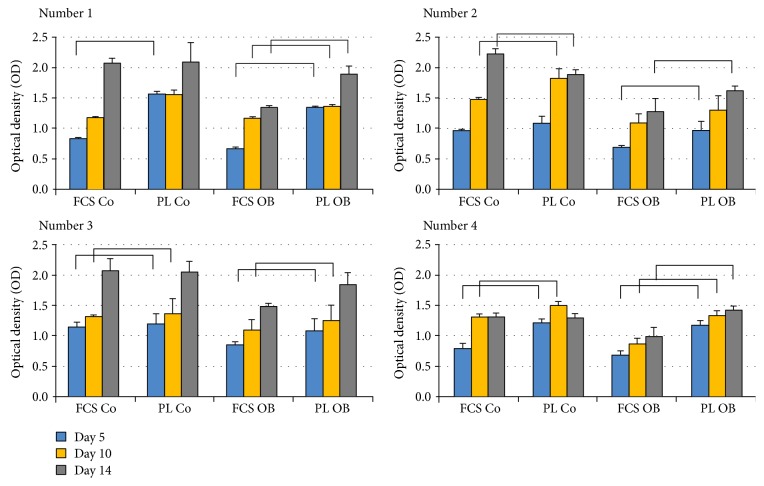
Measurements of metabolic JPC activities under FCS- and PL-containing culture conditions. JPCs from 4 donors (numbers 1–4) were cultivated under FCS and human PL supplementation under normal (Co) and osteogenic (Ob) conditions for 5, 10, and 20 days in 96-well plates for colorimetric measurements of metabolic activities. Optical densities (OD) are presented in the diagrams. The bars indicate statistical significances: *p* < 0.05.

**Figure 3:**
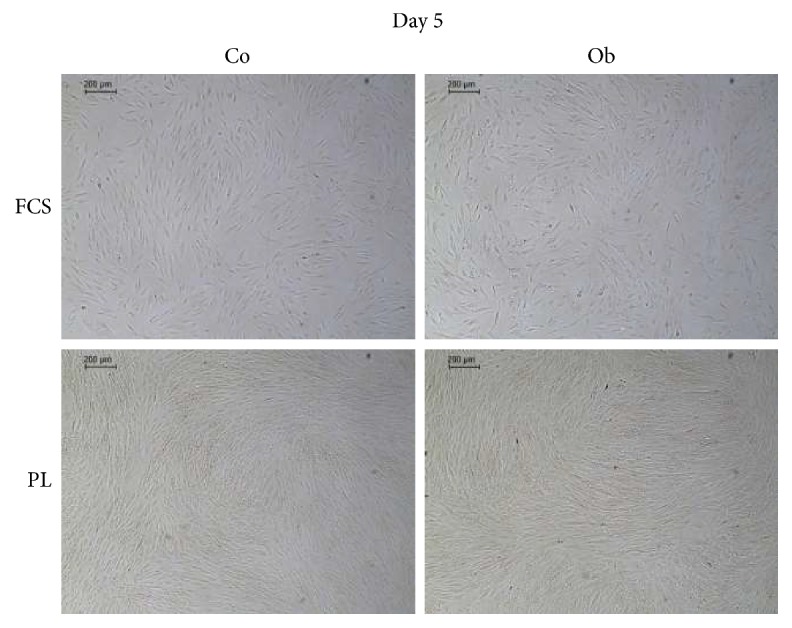
Representative microscopic images of FCS- and PL-cultured JPCs. JPCs were cultured under FCS or PL supplementation for 5 days and cell monolayers were visualized by microscopic images (scale bar: 200 *μ*m). Subsequently, cells were counted after trypsinization. Days 7 and 10 of *in vitro* culturing were also taken into account and growth rates and population doubling times were calculated, as listed in Tables 1 and 2.

**Figure 4:**
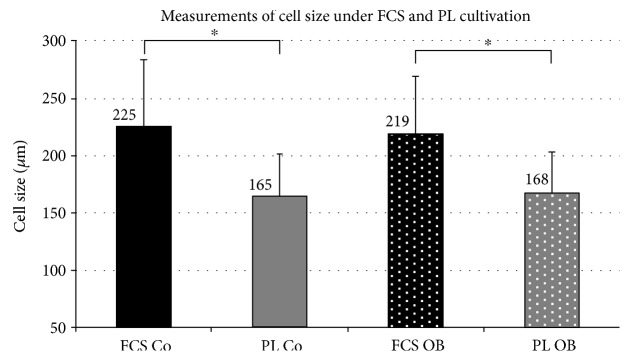
Measurements of cell size under FCS- and PL-containing culture conditions. Full length cell size measurements were performed using the CellProfiler and the LAS EZ software from 3 JPC donors (*n* = 10 measurements per donor, *n* = 30 for each culture condition). Cells were cultivated under FCS and human PL supplementation under normal (Co) and osteogenic (Ob) conditions for 5 days before performing the measurements. Statistical significances are indicated by asterisks: ^∗^*p* < 0.001.

**Figure 5:**
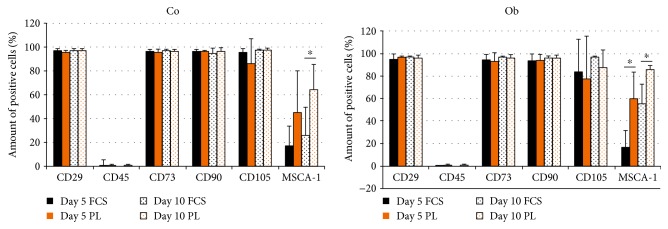
Flow cytometric analyses of MSC surface markers by JPCs cultivated under FCS and PL supplementation. Cell surface expression of CD29, CD45, CD73, CD90, CD105, and MSCA-1 was analyzed in JPCs (from 4 donors) cultivated under FCS and human PL supplementation under normal (Co) and osteogenic (Ob) conditions for 5 and 10 days by flow cytometry. Significantly higher levels (^∗^*p* < 0.05) of MSCA-1 were detected under Co conditions after day 10 and under osteogenic conditions after day 5 and 10 of hPL cultivation.

**Figure 6:**
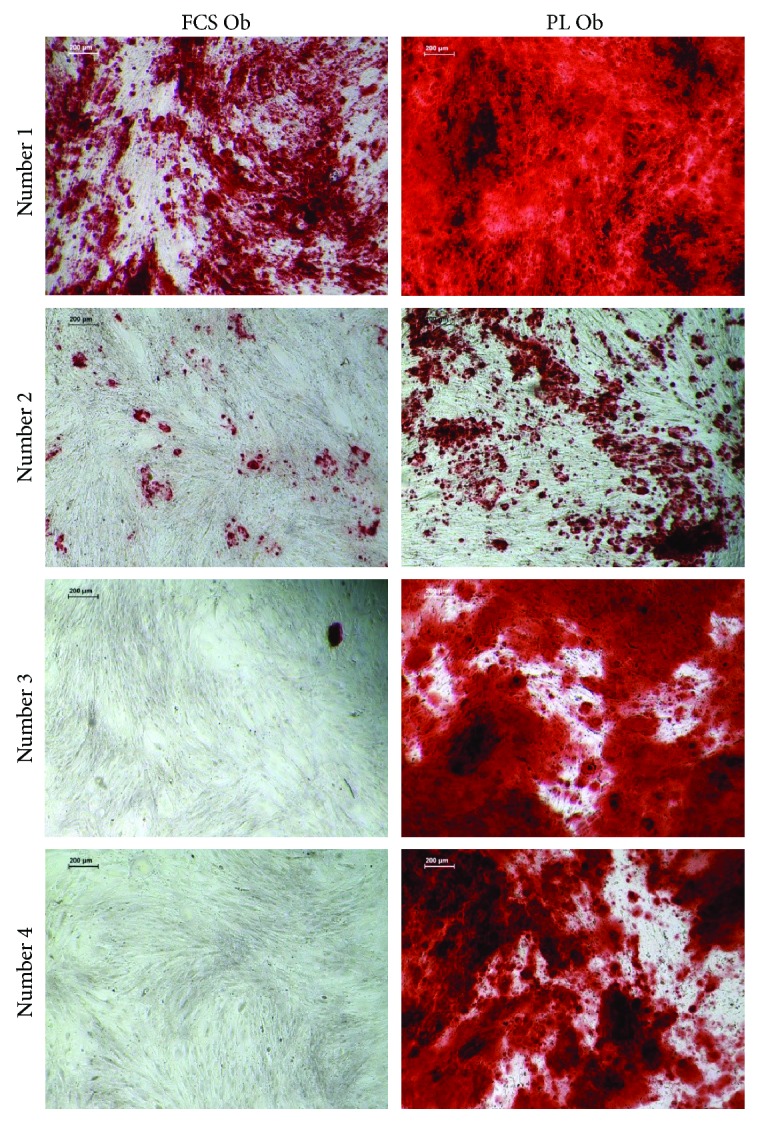
Detection of JPC mineralization cultured under FCS- and PL-containing culture conditions. Representative microscopic images (scale bar: 200 *μ*m) of alizarin red B stainings of PL- and FCS-cultured JPC monolayers (from 4 donors) after 24 days of osteogenic induction. Bright red-stained areas contain calcium phosphate precipitates.

Osteogenic conditions were performed for the experiments illustrated in Figures 1–6 by the addition of dexamethasone (4 *μ*M), *β*-glycerophosphate (10 mM), and L-ascorbic acid 2-phosphate (100 *μ*M). These osteogenic conditions are listed in the left column of Table 3, for other experiments, one or two of the osteogenic supplements were removed, as indicated in the table.

### 2.2. Live Monitoring of Cell Proliferation Using xCELLigence

Measurements of cell impedance were carried out using the RTCA DP analyzer (OLS OMNI Life Science, Bremen, Germany). Two hours before starting xCELLigence measurements, the device was put into the incubator for equilibration at 37°C. The required E-plates are equipped with gold-plated electrodes to enable noninvasive monitoring based on electrical impedance. After performing the resistor plate test, 100 *μ*l FCS or PL-containing (10%) DMEM/F12 medium was filled into the E-plates for background measurements. Plates were equilibrated for 2 hours before measurements. In the next step, 100 *μ*l containing 2000 cells was pipetted in each well of the E-plates. After half an hour of incubation at RT, live monitoring was started by a measurement interval of one hour. After an overnight incubation period, osteogenic induction was started. Medium change was performed every 2 days. For the calculation of population doubling times, data from 3 time points (day 5, 10, and 14) was extracted. The population doubling time is defined as the time span required for the doubling of the cell index and was calculated by the device-specific software.

### 2.3. Analysis of Cell Viability Using the Colorimetric EZ4U Assay

Cell viability measurements were performed using the EZ4U assay (Biomedica GmbH, Vienna, Austria) based on the conversion of tetrazolium salts in formazan derivates by the cell mitochondrial activity. End-point measurements were performed at 3 time points (day 5, 10, and 20) after adding 20 *μ*l of substrate to 200 *μ*l FCS/PL containing DMEM per well and a 4-hour incubation time. Optical densities were measured using the ELx800 plate reader (Biotek Instruments, Bad Friedrichshall, Germany) at a wavelength of 450 nm with a reference wavelength of 630 nm.

### 2.4. Counting of JPC Numbers Cultivated under PL- and FCS-Containing Culture Conditions and Calculation of Growth Rate and Population Doubling Time

JPCs were seeded in a density of 5 × 10^3^ cells/cm^2^, and cell counting was performed after 5, 7, and 10 days of PL and FCS culturing following trypsinization of JPCs (*n* = 5 donors, *n* = 4 counting per patient and culture condition). Based on the fact that osteogenically induced JPCs are not easy to separate to get single-cell suspensions, we chose for cell counting of untreated and osteogenically induced JPC early examination time points at day 5, 7, and 10 of in vitro culturing.

Counting was performed 4 times (per patient and culture condition, resp.). Growth rates (1) and population doubling times (2) were calculated using the following formula:

(1) $$\begin{array}{c} \mu =\frac{\ln\left(N_{t}/N_{0}\right)}{t}, \end{array}$$

(2) $$\begin{array}{c} t_{d}=\frac{\ln\left(2\right)}{t}, \end{array}$$

where *μ* is the growth rate (h^−1^), *t*_d_ is the population doubling time (h), *N*_0_ is the cell population at *t* = 0, *N*_*t*_ is the cell population at *t*, and *t* is time (h).

Growth rates and population doubling times from 5 patients were averaged and obtained mean values (±standard deviations) listed in Table 1 and Table 2, respectively.

### 2.5. Measurements of Cell Size of PL- and FCS-Cultured JPCs

Based on the visual impression of strongly diminished cell size under PL cultivation, measurements of full-length cell size using the CellProfiler and the LAS EZ software were performed. Therefore, FCS- and PL-cultured JPCs from 3 donors (*n* = 10 per donor) were used for untreated and osteogenic medium conditions (*n* = 30 for each culture condition).

### 2.6. Surface Antigen Expression Analysis by Flow Cytometry

PL- and FCS-cultured JPCs cultivated under undifferentiated and osteogenic conditions were detached from cell culture flasks and centrifuged (350*g* for 7 min), and pellets were resuspended in 20 ml of 10% Gamunex (human immune globulin solution, Talecris Biotherapeutics, Frankfurt, Germany) and incubated for 15 min at 4°C. The incubation with specific phycoerythrin- (PE-) labeled mouse anti-human CD29, CD45, CD73, CD90 (BD Biosciences Pharmingen, San Diego, USA), CD105 (AbD Serotec), and MSCA-1 (MACS Miltenyi Biotec, Bergisch Gladbach, Germany) in FACS buffer (PBS, 0.1% BSA, 0.1% sodium azide) followed, and cells were incubated for 15 min at 4°C. After two additional wash steps with FACS buffer, flow cytometric measurements with the Guava EasyCyte 6HT-2L instrument (Merck Millipore, Darmstadt, Germany) were performed.

### 2.7. Detection of Cell Mineralization by Quantification of Alizarin Dye Stainings

PL- and FCS-cultured JPCs from 5 donors were induced osteogenically (3 wells (of a 6-well plate) per culture condition) for 24 days, and cell monolayers were fixed with 4% formalin for 20 min. After two wash steps with PBS, 1 ml of a 40 mM alizarin dye solution with a pH of 4.2 was added to the monolayers for 20 min while shaking. Thereafter, the unbound dye was washed 4 times with dest water for 15 min. By adding of 10% acetic acid solution, alizarin dye was dissolved out from the monolayers for 20 min while shaking and cell layers were detached by scraping. After vigorous mixing and heating at 85°C for 10 min, samples were cooled on ice for 5 min and centrifuged at 20.000*g* for 20 min. Supernatants were neutralized by the addition of 10% ammonium hydroxide. Photometrical calcium quantification was then performed at a wavelength of 405 nm.

### 2.8. Statistical Analysis

For the evaluation of the data, means ± standard deviations are expressed, and for the statistical analysis, two-tailed Student's *t*-tests were used. A *p* value of <0.05 was considered significant.

## 3. Results

### 3.1. Measurements of JPC Population Doubling Times Using the Live-Monitoring System xCELLigence

JPCs were seeded into E-plate dishes and cell impedance was continuously evaluated under untreated (Co) and osteogenic (Ob) PL and FCS culture conditions. Based on the cell impedance data, population doubling (PD) times for three time points (day 5, 10, and 14) were calculated from the device-specific RTCA software 1.2.1. As individually illustrated (patient cell numbers 1, 2, 3, and 4) in Figure 1, we detected in almost all cases (with two exceptions: number 3 at day 14 and number 4 at day 10, both under undifferentiated (Co) conditions) significantly higher PD times in PL-cultured JPCs under both untreated (Co) and osteogenic (Ob) culture conditions. These data imply significant higher JPC proliferation activities under FCS culturing.

### 3.2. Measurements of Metabolic JPC Activities under PL- and FCS-Containing Culture Conditions

In contrast to the PD time measurements by cell impedance, we detected significantly higher metabolic activities of PL-cultured JPCs, as measured by the MTT-based assay and individually illustrated for the four analyzed patient cells in Figure 2. The resulted significant differences were detected both under undifferentiated (Co) and/or osteogenic culture conditions (Ob).

### 3.3. Counting of JPC Numbers Cultivated under PL- and FCS-Containing Culture Conditions

The abovementioned results obtained by x-CELLigence and the metabolic activity assay could not accurately reflect exact JPC proliferation rates.

By simple cell counting, we obtained significantly higher cell numbers when JPCs were cultured under PL supplementation under untreated and/or osteogenic conditions at all analyzed time points (day 5, 7, and 10). Representative microscopic images are shown in Figure 3. We detected highest proliferation rates after 5 days of culturing under PL supplementation (7-fold under untreated and 9-fold higher cell numbers under osteogenic conditions) compared to FCS supplementation. The differences between PL supplementation and FCS supplementation seemed, however, to become smaller at days 7 and 10 of in vitro cell culturing. Nonetheless, we counted on average 3-fold higher cell numbers under PL supplementation of untreated and osteogenically induced JPCs. Calculated growth rates and population doubling times (PDT) are listed in Tables 1 and 2. After 5 days of in vitro culturing, significantly higher PDTs (3-fold) were calculated for PL-cultured JPCs in comparison to FCS-cultured JPCs. After 7 days of in vitro culturing, 5-fold higher PDTs were obtained for untreated JPCs and 6-fold higher PDTs for osteogenically induced JPCs under PL supplementation. After 10 days of in vitro culturing again, 3-fold higher PDTs were calculated for PL-cultured JPCs compared to FCS-cultured JPCs. It is interesting to note the extremely high standard deviations for PDLs under FCS cultivation in contrast to the quite moderate deviations under PL supplementation.

### 3.4. Analysis of JPC Cell Size under PL- and FCS-Containing Culture Conditions

The results of PD times obtained by xCELLigence were quite contradictory to our observations made. By numerous cell countings and microscopic visualizations, the development of highly proliferating cells of significantly diminished cell sizes was observed under PL cultivation. Therefore, we performed measurements of JPCs derived from 3 patients (10 measurements per patient) under untreated/osteogenically induced PL- and FCS-containing culture conditions (*n* = 30 for each culture condition). As illustrated in Figure 4, significantly diminished cell sizes were detected under PL cultivation of both untreated and osteogenically induced cells. PL-cultured JPCs seemed to be on average 25% smaller than FCS-cultured cells and this difference in size achieved significant values under both culture conditions (FCS Co: 225.24 ± 58.59 versus PL Co: 165.11 ± 36.40; *p* < 0.001; FCS Ob: 219.08 ± 50.53 versus PL Ob: 167.65 ± 35.66; *p* < 0.001).

### 3.5. Flow Cytometric Analysis of Mesenchymal Cell Surface Marker Expression under PL- and FCS-Containing Culture Conditions

Mesenchymal stem cell marker expression was analyzed in untreated and osteogenic-induced JPCs (from 5 donors) under PL and FCS culture conditions. Averaged marker expression levels are illustrated in Figure 5. CD29, CD73, CD90, and CD105 were shown to be expressed at comparable levels by PL- and FCS-cultured JPCs under undifferentiated and osteogenically induced JPCs. All cells were almost completely negative for the leucocytic marker CD45.

Significant differences were detected for MSCA-1 cell surface expression. Under undifferentiated culture conditions, significantly higher MSCA-1 levels were detected only after day 10 of PL cultivation (Co day 5: FCS 16.82 ± 16.40 versus PL 45.12 ± 35.07; Co day 10: FCS 25.22 ± 23.78 versus PL 64.1 ± 21.45; *p* < 0.05). Under osteogenic culture conditions, a significantly higher degree of JPC positivity was detected at both analyzed time points for MSCA-1 expression under PL cultivation (Ob day 5: FCS 17.06 ± 14.70 versus PL 60.14 ± 23.48; *p* < 0.05; Ob day 10: FCS 55.32 ± 17.53 versus PL 86.32 ± 2.93; *p* < 0.05).

### 3.6. Analysis of the Mineralization Potential of JPCs Cultured under PL- and FCS-Containing Culture Conditions

**Figure 7:**
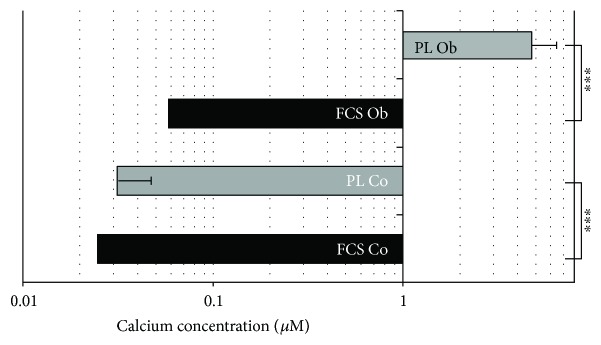
Calcium quantification of PL- and FCS-cultured JPCs. Alizarin dye was dissolved in acetic acid and colorimetric quantification (calcium concentration (*μ*M), logarithmic scale) was performed. Untreated and osteogenic induced JPCs under PL and FCS supplementation were considered. Statistical significances are indicated by asterisks: ^∗∗∗^*p* < 0.0001.

In Figure 6, representative microscopic images showed apparently higher amounts of calcium phosphate precipitates under PL (right panel) in comparison to FCS cultivation (left panel) of JPCs (4 donors). Subsequent quantification of alizarin red B stainings resulted in significant differences in calcium concentrations (*μ*M, logarithmic scale) under untreated as well as under osteogenic culture conditions (Co: FCS 0.025 ± 0.017 *μ*M versus PL 0.031 ± 0.015 *μ*M; Ob: FCS 0.058 ± 0.022 *μ*M versus PL 4.77 ± 1.74 *μ*M), as shown in Figure 7.

### 3.7. Analysis of JPC Mineralization under Different Osteogenic Induction Conditions

Table 3 shows the 5 different osteogenic medium conditions containing dexamethasone (dexa), *β*-glycerophosphate (*β*-glyc), and ascorbic acid (ascorb) in different combinations tested for mineralization induction of JPCs from 5 donors (numbers 1–5). Under FCS culture conditions, only 3 of 5 patient cells mineralized under the addition of all three osteogenic inducers to a relatively low extent. In contrast, PL-cultured JPCs mineralized all under these conditions and to a much higher extent. In addition, FCS-cultured cells derived from number 5 could also mineralize after addition of *β*-glyc and ascorbic acid.

Under PL cultivation, 2 of 5 patient cells mineralized strongly without the addition of ascorbic acid, whereas none of them mineralized under FCS cultivation upon addition of dexa and *β*-glyc. Most interesting was the mineralization capacity of JPCs under activation with *β*-glyc and ascorbic acid. 4 of 5 patient cells showed under PL cultivation a strong mineralization without the addition of dexamethasone. Additionally, JPCs derived from number 4 could also mineralize after the individual addition of only *β*-glyc.

## 4. Discussion

The use of FCS for the in vitro expansion of mesenchymal stromal cells for future cell therapies is restricted due to regulatory affairs and transmissions of animal diseases or causing immune reactions. To gain knowledge about the suitability of platelet lysate as a FCS substitute for the in vitro cultivation of jaw periosteal cells, we compared in the present study cell functions and mineralization potential of these cells under FCS and PL culturing. Since serum-free culturing of JPCs selectively promoted the osteoprogenitors within the whole cell population [1] and biochemical analysis of formed minerals elucidated their high quality by Raman spectrometry [2], the extent of mineralization was not satisfactory. To the best of our knowledge, we analyze for the first time in the present study the high potential of platelet lysate for the in vitro cultivation and mineralization of periosteal cells.

For the processing of human platelet lysates, several methods have been established, the most common of them being the implementation of one or more freeze/thaw cycles to induce platelet lysis and the release of abundant bioactive molecules [6]. However, a systematic analysis determining the optimal number of freeze/thaw cycles is still missing [3]. In a recent study, Bernardi and colleagues could demonstrate that the production method affects the efficacy of human PL for the expansion and differentiation of bone marrow-derived MSCs [7].

We analyzed cell surface marker expression as defined by Dominici and coauthors for MSCs [8]. All markers revealed comparable levels independent of FCS or PL cultivation. The only significant difference was referred to MSCA-1 surface expression which was detected at significantly higher levels in PL-cultured JPCs. We already demonstrated in a former study that the MSCA-1-positive cells hallmark the osteoprogenitors within the entire JPC cell population [9]. Growth factors derived from the used PL might trigger the proliferation of MSCA-1-positive cells and therefore be in part the reason for the much higher osteogenic potency of the herein analyzed JPCs.

Under PL cultivation, we observed and quantified a significantly diminished cell size. A similar observation was made under serum-free cultivation of JPCs [1]. This fact obviously impacts cell impedance measurements by altering the electrical resistance within the E-plates. The significantly higher PD times of FCS-cultured JPCs calculated by the xCELLigence software were shown to be in contrast to those obtained by measurements of the metabolic activities and by microscopic observations. These results indicate that this device is not suitable for the comparison of primary cells cultured at different media conditions which probably influence the cell size. Alternatively, the calculation of PD times by the device-specific software should be improved by including a correction factor considering the cell size.

Due to the fact that neither the xCELLigence system nor the MTT assay could accurately measure exact differences in cell proliferation under FCS- and PL-culturing, we performed simple cell counting and detected significantly higher cell numbers (3–9-fold) at all analyzed time points. Population doubling times were calculated to be 3-fold higher after 5 and 10 days and 5-6-fold higher after 7 days of in vitro culturing under PL cultivation of JPCs.

Numerous studies have proven the potency of hPL for the expansion of MSCs [10] from adipose tissue [11–13], umbilical cord blood, and bone marrow [14, 15]. In the present study, we proved additionally the potency of hPL for JPC proliferation and mineralization. An extensive comparative study examined in vitro proliferation of MSCs under the cultivation of 4 different media and three supplements including FCS, human AB serum, and human PL [16]. MSC proliferation was observed to be optimal in the PL-supplemented *α*MEM. The researchers assessed a library of soluble factors to promote robust MSC proliferation and established a cocktail which in combination with up to 1.2% PL could replace the addition of 5% hPL to the culture medium. The developed cocktail of recombinant human factors contained also hormones such as dexamethasone, insulin, and TSH. Fiorentini and coauthors demonstrated that the addition of dexamethasone ensures bone marrow MSC mineralization [17]. Interestingly, in our study, the addition of 10% hPL could replace the addition of dexamethasone to the osteogenic medium for robust mineralization of JPCs from 4 out of 5 tested donors indicating that the used hPL contains natural glucocorticoids that can replace dexamethasone. We further made the observation that *β*-glycerophosphate is essential and leads to a missing JPC mineralization when lacking as a phosphate source in the osteogenic medium.

A further interesting aspect might be the in vitro JPC culturing under hPL supplementation directly after the isolation from the primary tissue. In this study, frozen JPCs from passage 2 were thawed and expanded under FCS supplementation till sufficient cell numbers were achieved for the comparative experiments. The PL use from the starting point of JPC *in vitro* culturing could accelerate and improve the generation of tissue engineering constructs using this cell type.

In summary, we demonstrated in the present study the high potential of the supplementation with human platelet lysate instead of the commonly used FCS for the *in vitro* culturing and clinical tissue engineering applications of JPCs. Furthermore, the addition of the corticosteroid dexamethasone is probably no longer necessary after hPL supplementation.

## Figures and Tables

**Table 1:** Growth rate calculated from cell counting of FCS and PL-cultured JPCs under untreated and osteogenic culture conditions. 5 × 10^3^ cells/cm^2^ were seeded in 12-well culture plates and counted following trypsinization after 5, 7, and 10 days of in vitro culturing. Growth rate *μ* (h^−1^) calculated from total cell counts from 5 donors (*n* = 4 counting per patient and culture condition) is listed ±standard deviations and significance values.

	Growth rate *μ* (h^−1^)			
	FCS Co	PL Co	FCS Ob	PL Ob
d5	4.28*E*−03 ± 3.23*E*−03	1.58*E*−02 ± 1.48*E*−03	5.40*E*−03 ± 3.52*E*−03	1.91*E*−02 ± 2.77*E*−03
Significance	*p* < 0.01		*p* < 0.01	
d7	5.32*E*−03 ± 1.69*E*−03	2.41*E*−02 ± 4.46*E*−03	8.79*E*−03 ± 4.79*E*−03	3.42*E*−02 ± 2.36*E*−03
Significance	*p* < 0.01		*p* < 0.01	
d10	6.20*E*−03 ± 1.86*E*−03	2.20*E*−02 ± 2.93*E*−03	1.19*E*−02 ± 3.73*E*−03	3.00*E*−02 ± 2.88*E*−03
Significance	*p* < 0.01		*p* < 0.01	

**Table 2:** Doubling time *t*_d_ calculated from cell counting of FCS and PL-cultured JPCs under untreated and osteogenic culture conditions. 5 × 10^3^ cells/cm^2^ were seeded in 12-well culture plates and counted following trypsinization after 5, 7, and 10 days of in vitro culturing. Doubling time *t*_d_ (h) calculated from total cell counts from 5 donors (*n* = 4 counting per patient and culture condition) is listed ±standard deviations and significance values.

	Doubling time *t*_d_ (h)			
	FCS Co	PL Co	FCS Ob	PL Ob
d5	140.4 ± 55.5	44.2 ± 4.3	112.5 ± 39.2	37.2 ± 6.0
Significance	*p* < 0.05		*p* < 0.01	
d7	156.6 ± 84.6	29.9 ± 6.0	125.9 ± 104.8	20.4 ± 1.3
Significance	*p* < 0.05			
d10	125.6 ± 48.5	32.1 ± 4.3	66.5 ± 27.3	23.3 ± 2.1
Significance	*p* < 0.01		*p* < 0.05	

**Table 3:** JPC mineralization under FCS and PL cultivation by different combination of osteogenic stimuli. FCS- and PL-cultured JPCs from 5 donors (numbers 1–5) were induced by the addition of different combinations of osteogenic stimuli for 24 days before alizarin red stainings were performed.

Pat. number	FCS					hPL				
	dexa, *β*-glyc, ascorb	dexa, *β*-glyc	*β*-glyc, ascorb	dexa, ascorb	*β*-glyc	dexa, *β*-glyc, ascorb	dexa, *β*-glyc	*β*-glyc, ascorb	dexa, ascorb	*β*-glyc
1	+	0	0	0	0	+++	0	0	0	0
2	0	0	0	0	0	+	0	+++	0	0
3	0	0	0	0	0	+++	0	+++	0	0
4	+	0	0	0	0	+++	+++	+++	0	++
5	++	0	++	0	0	+++	+++	+++	0	0

dexa: dexamethasone; *β*-glyc: *β*-glycerophosphate; ascorb: ascorbic acid; 0: no mineralization occurred; +: low, ++: middle, +++: strong mineralization.
